# Toxicological Profile of a *Quercus laurina* Bark Extract Through Oral Administration: Impact on Hepatocyte Mitochondrial Bioenergetics

**DOI:** 10.1155/jt/8100007

**Published:** 2026-08-17

**Authors:** Grecia-Elena Hurtado-Nuñez, Sandra-Guadalupe Sánchez-Ceja, Lilia-Guadalupe Noriega, Sandra Tobón-Cornejo, José-Juan Virgen-Ortíz, Yoshio-de-Jesús Mena-Gavia, Isis-Beatriz Bermúdez-Camps, María-Carmen Bartolomé-Camacho, Héctor-Eduardo Martínez-Flores, Jorge-Armando Tinoco-Michel, Rosa-E. del Río, Martha-Estrella García-Pérez

**Affiliations:** ^1^ Faculty of Chemical-Pharmaceutical Biology, Michoacana University of San Nicolás de Hidalgo, Morelia, Michoacán, Mexico; ^2^ Department of Nutrition Physiology, National Institute of Medical Sciences and Nutrition Salvador Zubirán, Mexico City, Mexico, innsz.mx; ^3^ Center for Research in Food and Development, (CIAD, A.C.)-Center for Agro-Food Innovation and Development of Michoacán (CIDAM, A.C.), Morelia, Michoacán, Mexico; ^4^ Academic Area of Pharmacy, Institute of Health Sciences, Autonomous University of the State of Hidalgo, Pachuca de Soto, Mexico; ^5^ Institute of Chemical-Biological Research, Michoacana University of San Nicolás de Hidalgo, Morelia, Michoacán, Mexico

**Keywords:** glycolysis, hepatotoxicity, liver, mitochondria, oak

## Abstract

*Quercus laurina* is an oak species used orally in Mexican folk medicine. However, its toxicological profile has not yet been evaluated, which makes it difficult to determine safe doses for traditional preparations. This study, for the first time, evaluates the acute toxicity of an ethanolic extract of *Q. laurina* bark (QLBE) in Wistar rats in accordance with OECD guideline 423, and it explores the impact of QLBE on mitochondrial bioenergetics and glycolytic capacity in primary hepatocytes to determine whether the extract alters cellular energy metabolism. QLBE at 2000 mg/kg elicited death and tremors, and a dose‐dependent hepatic injury, in addition to histopathological changes in the kidney, brain, lung, and heart. QLBE caused a significant reduction in the basal, ATP‐linked, spare, and maximal mitochondrial respiratory capacities of hepatocytes, and it decreased glycolytic capacity after 2 and 24 h of exposure. These findings indicate that QLBE disrupts mitochondrial bioenergetics and glycolytic activity in hepatocytes, alterations that may be associated with the observed liver toxicity. HPLC and GC–MS analyses identified myo‐inositol, quininic acid, and ellagic acid as the primary metabolites of QLBE. The results demonstrated the toxic potential of QLBE, thereby discouraging its unregulated use in traditional remedies in Mexican folk medicine.

## 1. Introduction

The World Health Organization estimates that approximately 80% of the global population relies on medicinal plants or plant‐derived products as a primary source of healthcare [1]. In Mexico, the use of medicinal plants has been documented since pre‐Hispanic times and remains an important component of healthcare practices. According to the National Council for the Evaluation of Social Development Policy (CONEVAL), in 2024 approximately 50.2% of the Mexican population lacked social security, and 39.1% had no access to health services [2]. Consequently, many people rely on alternative forms of medicine, such as medicinal plants, which are commonly recommended by “yerberos,” traditional healers, and even health professionals, many of whom support receiving academic training on the appropriate prescription and use of medicinal plants [3].

Despite their widespread use, the pharmacological, phytochemical, and toxicological information available for many plants employed in traditional Mexican medicine remains limited [3]. This lack of scientific evidence hinders the rational use of medicinal plants and their evidence‐based integration into the healthcare system.

Species of the genus *Quercus* represent the second most commonly used botanical genus in Mexico due to the medicinal properties attributed to their bark [4]. This prominent role in Mexican herbal medicine may be explained by the wide geographic distribution of these species across the country, as Mexico represents the second global center of diversity for this genus, harboring 161 species, 109 of which are endemic [5].

In Mexico, barks from *Quercus* species are commonly sold in local markets [4] and through online commercial platforms such as Amazon and “Mercado Libre,” where oak bark is marketed at prices ranging from 149 to 265 MXN per kilogram (around 15 USD per kg). However, this largely unregulated trade raises important public health concerns, as the specific species being commercialized is often unknown and toxicological information required to establish safe dosages in traditional preparations is generally lacking. Consequently, there is an urgent need to characterize the safety profile of extracts derived from oak bark used in traditional Mexican medicine.

*Quercus laurina* Humb. & Bonpl., commonly known as “laurelillo oak,” “chilillo oak,” or white oak, is a species native to Mexico whose bark has been traditionally used to treat postpartum complications, bladder dysfunction, urinary tract infections, bleeding gums, and various skin infections [6–8]. The ethanolic extract of *Q. laurina* bark (QLBE) exhibits significant antioxidant activity against biologically relevant reactive species, including nitric oxide and hypochlorous acid [9], suggesting potential therapeutic relevance in the management of oxidative stress‐related disorders. However, despite its promising biological activity, the toxicological profile of QLBE remains unknown, representing a critical gap that limits its safe use and potential therapeutic application.

Previous investigations have demonstrated discrepancies in the toxicological characteristics of extracts derived from oak species, including *Quercus sideroxyla* [10], *Quercus branti* [11], *Quercus suber* [12–14], *Quercus leucotrichophora* [15], *Quercus dilatata* [16], *Quercus ilex* [17], *Quercus infectoria* [18], *Quercus serrata* Murray [19], and *Quercus crassifolia* [20]. These findings suggest that the toxicological properties of *Quercus* species may vary considerably among species, highlighting the need for species‐specific safety evaluations.

Understanding the mechanisms underlying the potential toxicity of plant extracts is therefore essential. Among the cellular targets involved in xenobiotic toxicity, mitochondria play a central role in maintaining cellular energy metabolism and redox homeostasis [21]. Mitochondrial dysfunction has been recognized as a key event in the development of drug‐induced and xenobiotic‐induced toxicity [22], particularly in metabolically active organs such as the liver. Accordingly, evaluating the effects of plant‐derived extracts on mitochondrial bioenergetics and glycolytic capacity represents a valuable strategy for identifying early toxicological events at the cellular level.

Despite the traditional use of its bark and the reported antioxidant activity of *Q. laurina*, the toxicological profile of this species remains largely unexplored. Therefore, the aim of the present study was to evaluate, for the first time, the acute oral toxicity of the QLBE in Wistar rats and to investigate its impact on mitochondrial bioenergetics and glycolytic capacity in primary hepatocytes. This integrated approach provides putative mechanistic insights into the cellular effects of QLBE and contributes to the scientific evaluation of its safety, supporting the rational use of this species in traditional medicine.

## 2. Materials and Methods

### 2.1. Obtention of *Q. laurina* Bark Extract

The bark of *Q. laurina* was collected in a forest plantation in Puente de Tierra, Ciudad Hidalgo, Michoacán (latitude 19°30′28.3″ N, longitude 100°42′45.0″ W). The botanical specimen was deposited in the EBUM Herbarium of the Faculty of Biology, UMSNH, under the voucher number [18789], to ensure proper documentation and traceability of the botanical material.

The oak bark was washed, fractionated, and dried at 40°C for 48 h until its moisture content was < 10%. After drying, the material was crushed using a Thomas Digital ED‐5 Wiley cutting mill (Swedesboro, NJ, USA) and sieved through a #40 mesh (400 μm) to achieve a uniform particle size.

*Q. laurina* bark was extracted by maceration as previously reported [23]. To obtain sufficient extract for all experimental procedures, four separate macerations were performed, each using 20 g of powdered *Q. laurina* bark and 200 mL of 90% ethanol, with shaking at 220 rpm for 48 h at 22°C under light‐protected conditions. The resulting liquid extracts were filtered through Whatman 42 filter paper, and the residual solids were washed with an additional 200 mL of 90% ethanol. The filtrates from the four independent macerations were then pooled and concentrated using a rotary evaporator (BUCHI Rotavapor R‐300, Fisher Scientific, Waltham, MA, USA) to remove the solvent. The remaining aqueous residue was lyophilized (FreeZone 2.5 L Benchtop Freeze Dry System, Labconco, Kansas City, MO, USA), and the resulting solid extract was stored in amber vials at 4°C. This procedure yielded a sufficient sample for HPLC and GC–MS analyses, elemental characterization, in vivo acute toxicity tests, and in vitro experiments with primary hepatocytes. The extraction yield was calculated as shown in (1).

(1) % sample yield=W1×100W2,

where *W*_1_—weight of dry extract and *W*_2_—weight of dry bark powder.

### 2.2. Chemical Characterization

#### 2.2.1. HPLC Analysis of Phenolic Compounds

Analysis of individual phenolic compounds was performed using an HPLC system (1260 Infinity, Agilent Technologies Inc., Santa Clara, CA, USA). The HPLC was equipped with a quaternary pump unit, a diode array detector, an automatic sample injector, and a column oven. Separation of phenolic compounds was performed at 35°C on an InfinityLab Poroshell 120 EC‐C18 column (4 μm particle size, L × ID: 250 × 2.1 mm) (Agilent). The samples were eluted with a gradient of 1.0% aqueous formic acid (A) and acetonitrile (B) at a flow rate of 0.5 mL/min. The gradient was as follows: the mobile phase was maintained at 2% B for 5 min, then changed linearly to 10% B at 15 min, 40% B at 30 min, and 55% B at 45 min and held for 5 min, followed by re‐equilibration for 15 min. The procedure for preparing the sample and separating the phenolic compounds was performed as described by Schieber et al. [24]. The dry QLBE was prepared in methanol at a concentration of 5 mg/mL. The resulting supernatant was filtered through a 0.45 μm PTFE filter (Agilent Technologies, Germany) and transferred to 2 mL amber vials for HPLC analysis. A volume of 5 μL was injected, and the phenolic compounds were monitored at *λ* = 255, 260, 270, 310, 322, 355, and 370 nm. The following fifteen phenolic compound standards, with a purity of ≥ 98% from Sigma‐Aldrich, were used to establish calibration curves: gallic acid, 3,4‐dihydroxybenzoic acid, 3‐hydroxytyrosol, 4‐hydroxybenzoic acid, vanillic acid, caffeic acid, chlorogenic acid, syringic acid, 4‐hydroxy‐3‐methoxybenzaldehyde, p‐coumaric acid, p‐ferulic acid, sinapic acid, ellagic acid, rutin, and quercetin. Chromatographic peaks in samples were identified as phenolic compounds by comparing their retention times with those of corresponding analytical standards, analyzing their UV spectra, and spiking samples with an appropriate standard.

#### 2.2.2. Elementary Analysis

The elemental composition of QLBE, including its carbon (C), hydrogen (H), nitrogen (N), and sulfur (S) contents, was determined using a Leco TruSpec Micro elemental analyzer (series 628; St. Joseph, MI, USA). The oxygen content was calculated by difference, considering the ash content on a dry basis.

#### 2.2.3. GC–MS Analysis

Major molecules in QLBE were identified by GC–MS using an Agilent GC 7890B system with a 10 μL injector, a 5181‐3354 7000 GC/MS Triple Quad (Santa Clara, CA, USA), and a HP‐5MS UI 60°C–325°C, 30 m × 0.250 mm × 0.25 μm column. Because derivatization before GC–MS analysis reduces the polarity of target molecules such as phenols, thereby increasing their volatility, the extract was analyzed by GC–MS after derivatization. For derivatization, 5 mg of *Q. laurina* bark extract was mixed with 200 μL pyridine (Sigma‐Aldrich CAS 110‐86‐1) and 200 μL N,O‐bis(trimethylsilyl)trifluoroacetamide (BSTF) containing 1% trimethylchlorosilane (TMCS) (Sigma‐Aldrich CAS 25561‐30‐2). The mixture was stirred, heated in an oven at 70°C for 2 h, and cooled at 4°C for 15 min. The sample was then diluted with 400 μL of ethyl acetate, centrifuged at 4500 rpm for 8 min, and the supernatant was transferred to a new GC vial [25]. The molecules were identified by comparing their mass spectral fragmentation and retention times with standard reference spectra generated from the mass spectral library using Mass Hunter Qualitative Analysis B.07.00 and NISS 2.2 Mass Spectral Search software. The data obtained were plotted in OriginPro (2022) (Northampton, MA, USA).

### 2.3. Acute Toxicity Assessment

After the chemical characterization of the QLBE was conducted, an acute oral toxicity test was performed. This study was initiated due to the lack of existing toxicological data on the species and the significant variation in the toxicological properties of *Quercus* species. The acute oral toxicity assessment of the QLBE was conducted in accordance with OECD Test Guideline No. 423 (Acute Toxic Class Method) [26]. Experiments were conducted in compliance with Mexican regulations on the use and care of experimental animals (NOM‐062‐ZOO‐1999, Mexico) [27], the ARRIVE guidelines [28], and the National Institutes of Health Guide for the Care and Use of Laboratory Animals [29] and were approved by the Research Ethics Committee, Research and Biosafety of the Michoacana University (CEIIB‐UMSNH: 254/2025).

#### 2.3.1. Animals

The 8‐week‐old, healthy, nulliparous, nonpregnant female Wistar rats were obtained from the UNAM Bioterium’s Juriquilla campus in Querétaro, Mexico. Acute toxicity tests are often performed on only one sex. It is generally assumed that females are more sensitive than males to the acute toxic effects of chemicals. This difference is attributed to hormonal and reproductive factors, consistent with the Organization for Economic Cooperation and Development (OECD) Test Guideline 423 [26]. The animals were housed at 22°C ± 3°C for 7 days, with humidity between 30% and 70%. The setting was artificially lit with 12‐h light–dark cycles. Water and food were available *ad libitum.*

#### 2.3.2. Experimental Design and Treatment

Wistar rats were randomly assigned to groups of three for each experimental step to evaluate doses of 50, 300, and 2000 mg/kg body weight (bw). Animals were fasted for 8 h prior to administration and had free access to water. On the day of administration, the QLBE was dissolved in purified water to prepare stock solutions at concentrations of 5, 20, and 150 mg/mL, corresponding to the 50, 300, and 2000 mg/kg dose levels, respectively. All doses were administered in a standardized volume not exceeding 2 mL/100 g bw [26]. Following oral administration, animals were observed for signs of toxicity and mortality. Initial observations were conducted within the first 30 min, followed by hourly assessments for the next 12 h, and then daily evaluations for 14 days.

Since no previous information was available on the acute oral toxicity of QLBE, the study was conducted in accordance with OECD Guideline 423 (Acute Toxicity Class Method; Annex 2c) [26]. The evaluation began with the administration of 300 mg/kg to an initial group of 3 rats. Due to the absence of mortality or severe signs of toxicity, a confirmatory test was conducted with 3 additional animals at the same dose (*n* = 6) as stated in Guideline 423 [26].

Subsequently, a dose of 2000 mg/kg bw was evaluated in a group of three animals, where the death of one animal was recorded within the first 12 h following administration. In accordance with the sequential design established in Annex 2c [26], a second group of three animals was treated with the same dose (2000 mg/kg bw) to confirm the observed toxicological response, resulting in two additional deaths. In total, three of the six animals treated with this dose died during the observation period, while the three surviving animals were included in subsequent analyses.

Because no mortality was observed at 300 mg/kg bw, the 50 mg/kg dose was subsequently administered to a group of three animals (*n* = 3). A control group consisting of three rats was maintained throughout the experiment (*n* = 3).

To minimize the use of laboratory animals and in accordance with the 3Rs principle (replacement, reduction, and refinement), the number of animals was strictly limited to those necessary for the toxicological evaluation as specified by the Acute Toxicity Class Method [26, 30].

Overall, a total of 18 animals were used in the study, of which 15 survived until the end of the experimental period. Consequently, for biochemical, hematological, histopathological, and body weight analyses, only these surviving animals were included, as it was not possible to obtain biological samples from the animals that died during the post‐administration period. For the deceased rats, organs were removed during necropsy to determine the cause of death.

Body weights of surviving animals were measured on Days 0, 7, and 14. On Day 14, animals were anesthetized with sodium pentobarbital (50 mg/kg bw) by intraperitoneal injection. Blood was collected via cardiac puncture, and organs were preserved for subsequent analysis.

#### 2.3.3. Biochemical and Hematological Determinations

Biochemical and hematological markers were determined at the Edulab Laboratory in Morelia, Michoacán, Mexico. Before analyzing the samples, protocols were established for handling rat plasma and serum. The hematological evaluation was performed using electrical impedance with Abbott’s CELL‐DYN Emerald instrument (Abbott Park, Illinois, USA), and blood chemistry parameters were assessed using a colorimetric method with the Cobas c111 analyzer from Hoffman‐La Roche Ltd (Basel, Switzerland). Serum electrolytes were measured using the ion‐selective electrode feature of the AVL 9180 analyzer, also from Hoffman‐La Roche Ltd (Basel, Switzerland).

#### 2.3.4. Macroscopic and Histopathological Analysis

Macroscopic observations were used to evaluate the structure, appearance, and location of the brain, heart, lungs, kidneys, adrenal glands, liver, pancreas, spleen, stomach, large intestine, and small intestine. Their relative organ weights were calculated using the following equation:

(2) relative organ weight %= organ weightbody weight at the time of sacrifice×100.

For histopathological analysis, tissues were fixed in neutral buffered formalin at pH 7.4, dehydrated through a graded series of ethanol (60%, 70%, 80%, 90%, and 100%), and cleared in xylene. The tissues were embedded in paraffin and sectioned at 3 μm using a Leica RM2125 RTS manual microtome (Deer Park, Illinois, USA). The slides were stained with hematoxylin and eosin according to standard procedures. Masson’s trichrome staining was performed on liver sections to detect fibrosis. Three slides with three serial sections each were prepared from each tissue block per rat. A total of nine sections per organ were analyzed in each rat. In each section, uniform, random, systematic sampling was conducted by scanning the entire tissue section. For each tissue, 100 randomly selected, non‐overlapping fields of view were analyzed. This approach ensured that all parenchyma areas had an equal chance of being evaluated, thereby avoiding selection bias [31]. The histopathological index was evaluated in a blinded manner by two qualified pathologists. Discrepancies were resolved by consensus to ensure agreement in the interpretation of histological findings. The histopathological scoring of liver lesions was based on previous research with some modifications, as detailed in the supporting information (Table 1S) [32, 33]. For renal scoring, the standards described by Liu et al. [34] were used, with some modifications as detailed in the supporting information (Table 2S). Tissue sections were analyzed and captured using a brightfield microscope equipped with a Carl Zeiss AxioCamMRC digital video camera (Munich‐Hallbergmoos, Germany).

### 2.4. Impact of QLBE on Mitochondrial Function

Further evaluated the effect of the QLBE extract on mitochondrial function in primary mouse hepatocytes using the extracellular flux analyzer technology (Seahorse XFe96). This technique accurately measures the oxygen consumption rate (OCR) and the extracellular acidification rate (ECAR) of living cells in real time [35]. Primary hepatocytes were obtained from male C57BL/6 mice aged 8–12 weeks, provided by the Experimental Research Department and Animal Care Facility at the National Institute of Medical Sciences and Nutrition Salvador Zubirán (INCMNSZ), as previously described [36]. The experimental protocol was approved by the Animal Care and Ethics Committee of the National Institute of Medical Sciences and Nutrition Salvador Zubirán (CICUAL‐FNU‐2002‐20‐22‐1). Briefly, in situ liver perfusion with collagenase was performed following the method of Berry and Friend [37]. The isolated liver was then disaggregated in Hank’s balanced salt solution (HBSS), and the suspension was strained through a 70 μM mesh. The filtered cells were washed twice with HBSS and resuspended in D‐MEM/F‐121 (Gibco, Grand Island, NY, USA) supplemented with 10% fetal bovine serum (FBS) and 1 × antibiotic.

Mouse primary hepatocytes were seeded at a density of 4000 cells/well in an XFe96 microplate. Cell density was determined by performing a cell density curve to ensure that OCR and ECAR measurements were obtained within the linear detection range. After 4 h, the medium was changed to remove unattached cells, and hepatocytes were then treated with 0, 50, 100, 200, 500, 1000, and 1500 μg/mL of the QLBE extract for 2 or 24 h. Then performed a mitochondrial stress test using an XFe96 extracellular flux analyzer (Agilent Technologies, Santa Clara, CA, USA).

Briefly, cells were washed and incubated for 1 h in a non‐CO2 incubator (Seahorse XF) with Dulbecco’s Modified Eagle Medium (DMEM) at pH 7.4, supplemented with 1 mM pyruvate, 2 mM glutamine, and 10 mM glucose. During the experiment, 2 μM oligomycin, 1 μM carbonyl cyanide p‐(trifluoromethoxy)phenylhydrazone (FCCP), 1 μM rotenone/antimycin A (R/A), and 50 mM 2‐deoxyglucose (2‐DG) were sequentially injected, and three OCR and ECAR measurements were performed under basal conditions and after each addition. Hoechst 33342 (2 μM) was co‐injected with the 2‐DG to dye the nucleus. At the end of the assay, cell nuclei were counted using Gen5 and Cytation 1 (BioTek). OCR and ECAR measurements were normalized to the number of cells. Basal mitochondrial respiration, ATP‐linked respiration, proton leak, maximal respiration, spare respiratory capacity, and nonmitochondrial respiration were calculated from the OCR values. Glycolysis, glycolytic capacity, glycolytic reserve, and nonglycolytic acidification were calculated from the ECAR values as previously described [38].

### 2.5. Statistical Analysis

The experimental results are presented as mean ± standard error of the mean (SEM) considering the surviving animals from the administered doses. Normality was tested using the Shapiro–Wilk test, and homogeneity of variance was tested using Levene’s test. The Kruskal–Wallis test was used for body weight, hematological, and biochemical analyses, followed by the Wilcoxon and Dunn’s post hoc tests. Hepatic and renal histopathologic scores are expressed as mean ± SEM and were determined using the Kruskal–Wallis one‐way ANOVA, followed by Dunn’s multiple comparison post hoc test. Differences were considered statistically significant at *p* < 0.05. Mitochondrial function assay results are presented as mean ± SEM. The mitochondrial stress assay was repeated to ensure reproducibility and analyzed by one‐way ANOVA, followed by Dunnett’s post hoc test to compare with cells treated with vehicle alone (0 μg/mL). Data analysis was performed using JMP Pro 16.0.0 (SAS Institute, Raleigh, NC) and GraphPad Prism 8 (San Diego, CA, USA).

## 3. Results

### 3.1. Chemical Characterization

The recovery of the QLBE was 16.84 ± 3.45% w/w relative to the dried bark. HPLC–DAD analysis of QLBE confirmed the presence of four phenolic compounds, including ellagic acid, gallic acid, 3,4‐dihydroxybenzoic acid, and t‐ferulic acid, out of the fifteen analyzed compounds (Figure 1, Table 1).

**FIGURE 1:**
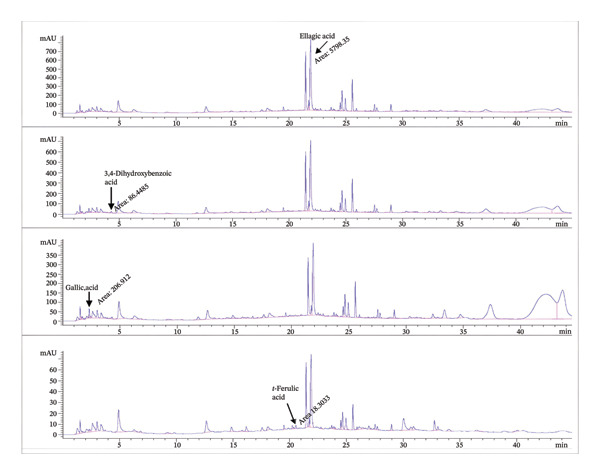
HPLC–DAD chromatograms of phenolic compounds identified in the ethanolic extract of *Q. laurina* bark.

**TABLE 1:** Phenolic compounds analyzed by HPLC‐DAD in *Q. laurina* bark extract.

Phenolic compound	Retention time (min)	*λ*_max_ (nm)	Presence
Gallic acid	2.30	270	Yes
3,4‐Dihydroxybenzoic acid	4.31	260	Yes
3‐Hydroxytyrosol	4.69	275	ND
4‐Hydroxybenzoic acid	7.50	255	ND
Vanillic acid	11.96	260	ND
Caffeic acid	12.91	322	ND
Chlorogenic acid	13.28	322	ND
Syringic acid	15.02	275	ND
4‐Hydroxy‐3‐methoxybenzaldehyde	15.82	275	ND
*p*‐Coumaric acid	17.725	310	ND
*t*‐Ferulic acid	20.539	322	Yes
Sinapic acid	21.325	322	ND
Ellagic acid	21.904	255	Yes
Rutin	22.197	355	ND
Quercetin	26.871	370	ND

Table 2 presents the elemental analysis of QLBE. This extract was particularly rich in carbon and oxygen, with a low nitrogen content.

**TABLE 2:** Elementary analysis of *Quercus laurina* bark extract.

*Quercus laurina* extract	
Elementary analysis	(wt.%)
C^b^	50.33 ± 0.13
H^b^	4.91 ± 0.01
N^b^	0.07 ± 0.00
O^c^	44.69 ± 0.14

*Note:* Carbon (C), hydrogen (H), nitrogen (N), sulfur (S), and oxygen (O). Data represent the mean ± SD of two independent experiments.

^b^Dry basis.

^c^By difference.

Figure 2 shows the GC–MS chromatogram of QLBE, and Table 3 lists the identities of its major compounds. A total of 21 molecules were identified, including organic acids (9.5% of the total), alcohols (9.5%), cyclitols (19%), sugars (43%), phenols (9.5%), and fatty acids (9.5%). Myo‐inositol (3) and quininic acid (7) were the most abundant compounds.

**FIGURE 2:**
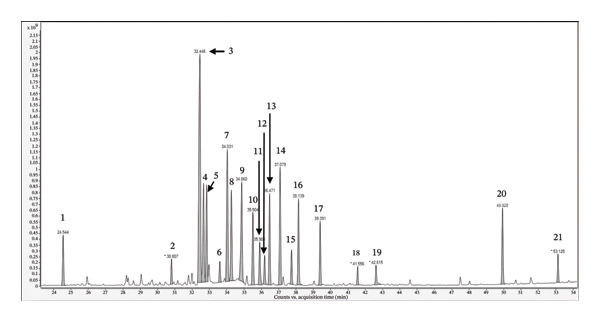
GC–MS analysis of the ethanolic extract of *Q*. *laurina* bark.

**TABLE 3:** Molecules identified in *Q. laurina* bark extract according to GC–MS analysis.

No.	Compound	RT (min)	Area (%)	Group
1	Malic acid	24.54	2.92	AC
2	Xylitol	30.81	1.24	AL
3	Myo‐Inositol	32.45	19.96	Cyc
4	D‐(−)‐Fructofuranose, pentakis(trimethylsilyl) ether (isomer 1)	32.66	6.50	S
5	D‐(−)‐Fructofuranose, pentakis(trimethylsilyl) ether (isomer 2)	32.83	5.66	S
6	D‐(+)‐Talofuranose, pentakis(trimethylsilyl) ether (isomer 2)	33.60	1.21	S
7	Quininic acid	34.03	9.36	AC
8	D‐Arabinopyranose (isomer 1)	34.26	5.63	S
9	β‐D‐(+)‐Mannopyranose	34.86	7.43	S
10	Inositol, epi‐	35.50	4.51	Cyc
11	D‐Sorbitol	35.90	2.66	AL
12	Gallic acid	36.19	1.67	Ph
13	D‐Ribopyranose	36.47	5.36	S
14	β‐D‐Glucopyranose	37.08	7.37	S
15	Palmitic Acid	37.74	1.89	FA
16	Scyllo‐Inositol	38.14	4.98	Cyc
17	D‐Ribose	39.39	3.52	S
18	Stearic acid	41.56	0.97	FA
19	Galactinol, nonakis(trimethylsilyl) ether	42.62	1.30	Cyc
20	Sucrose	49.93	4.38	S
21	Catechine, (2*R*‐*cis*)‐,	53.13	1.48	Ph

*Note:* AC, acids; Cyc, cyclitol; S, sugar; Ph, phenol; AL, alcohol.

Abbreviation: FA, fatty acid.

### 3.2. Oral Acute Toxicological Assessment

#### 3.2.1. Signs of Toxicity, Body Weight, and Relative Organ Weight

Clinical signs of toxicity were observed in the group treated with 2000 mg/kg bw, including piloerection, drowsiness, lethargy, tremors, loss of balance, and ocular abnormalities (opacity and exophthalmos). No signs of toxicity were detected in the 300 mg/kg or 50 mg/kg bw groups. Table 4 shows the body weights (g) of the Wistar rats recorded on Days 0, 7, and 14 of the study. There were no statistically significant differences in body weight (*p* < 0.05).

**TABLE 4:** Body weight progression in animals receiving oral administration of QLBE.

	Control	50 mg/kg	300 mg/kg	2000 mg/kg
Day 0	226.67 ± 1.45^ab^	221.33 ± 0.88^b^	242.83 ± 3.91^a^	239.17 ± 5.41^ab^
Day 7	240.33 ± 3.18^ab^	230.67 ± 2.40^b^	255.83 ± 4.28^a^	241.00 ± 2.08^ab^
Day 14	248.33 ± 4.06^ab^	240.00 ± 4.04^b^	261.33 ± 5.88^a^	257.00 ± 2.31^ab^

*Note:* Values represent the mean ± SEM. Wilcoxon with Dunn’s post hoc test, (*p* < 0.05). Control (*n* = 3); 50 mg/kg (*n* = 3); 300 mg/kg (*n* = 6); 2000 mg/kg (*n* = 3). Different letters in the same column indicate statistically significant differences.

Figure 3 shows the relative weights of the target organs. Administration of QLBE did not significantly affect the relative weights of the organs. However, at 2000 mg/kg, there was a trend toward increased weights of the right and left kidneys and the right and left lungs.

**FIGURE 3:**
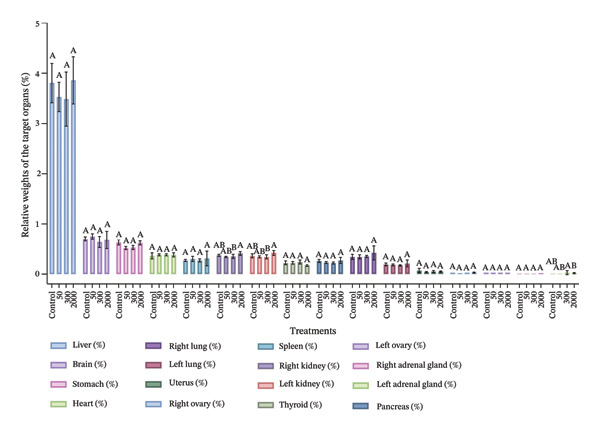
Relative weights of the target organs of Wistar rats orally administered *Q. laurina* bark extract. Values are expressed as mean ± SEM relative to the control group. Different letters indicate statistically significant differences (Wilcoxon with Dunn’s post hoc test, *p* < 0.05).

#### 3.2.2. Biochemical and Hematological Analysis

The hematopoietic system is recognized as a target for toxic agents. Hematological analysis following oral administration of QLBE is presented in Figure 4. A notable increase in total leukocyte count was observed at 2000 mg/kg compared with the control (*p* < 0.05). Additionally, there was a trend toward increased platelet and monocyte levels at the same dose.

**FIGURE 4:**
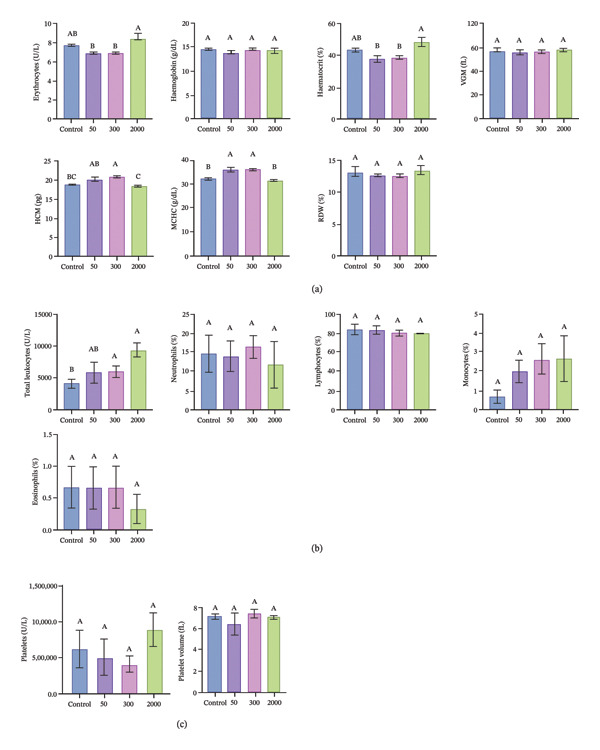
Hematological parameters after oral administration with *Q. laurina* bark extract. Values are expressed as mean ± SEM for the control group. Different letters indicate statistically significant differences (Wilcoxon with Dunn’s post hoc test, *p* < 0.05). (a) Red blood cell series; (b) white blood cell series; (c) platelets. MCV, mean corpuscular volume; MCH, mean corpuscular hemoglobin; MCHC, mean corpuscular hemoglobin concentration; RDW, red cell distribution width.

Biochemical evaluations indicated no significant differences between the QLBE‐treated and control groups Figure 5. However, a downward trend was observed in parameters such as urea levels in a dose‐dependent manner relative to the control group, whereas AST, ALT, and creatinine levels declined at 2000 mg/kg. In contrast, at the highest dose, alkaline phosphatase levels tended to increase in the QLBE‐administered groups compared with the control group, as did lactate dehydrogenase and bilirubin.

**FIGURE 5:**
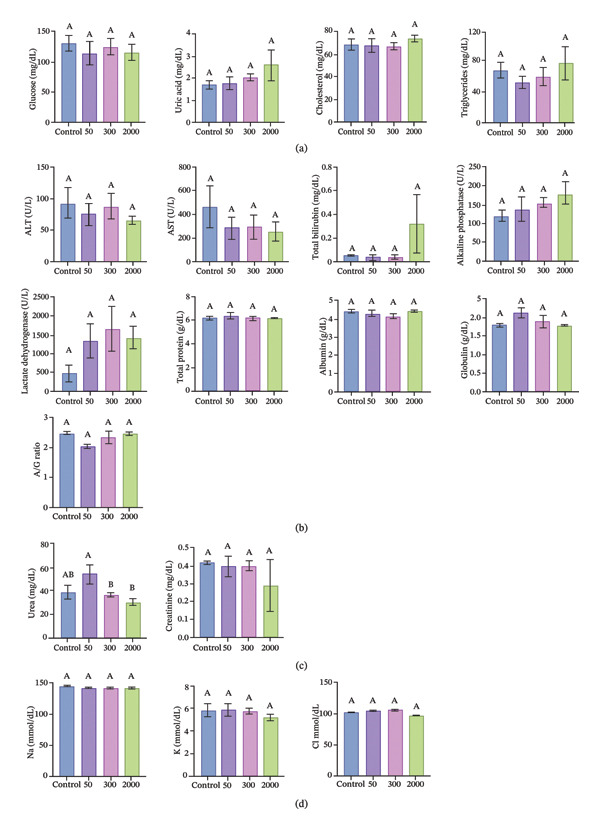
Biochemical parameters after oral administration with *Q. laurina* bark extract. Values are expressed as mean ± SEM for the control group. Different letters indicate statistically significant differences (Wilcoxon with Dunn’s post hoc test, *p* < 0.05). (a) Lipid, carbohydrate, and purine metabolism; (b) hepatic function and proteins; (c) renal function; (d) electrolytes. ALT: Alanine aminotransferase, AST: aspartate aminotransferase.

### 3.3. Histopathological Analysis

#### 3.3.1. Liver Pathology Score

Histological examination of the liver revealed dose‐dependent hepatic injury in the groups administered QLBE Figure 6a. As expected, the control group exhibited a normal appearance of the hepatic parenchyma, centrilobular veins, and portal triads (Figure 6a–a1, b1, c1, d1). At 50 mg/kg, findings included endothelial detachment and rupture within centrilobular veins, infiltration of polymorphonuclear leukocytes (PMNs), dilation of sinusoidal spaces, peliosis, and fragmentary coagulative necrosis (Figure 6a–a2, b2, c2, d2). Rats administered 300 mg/kg exhibited endothelial detachment and rupture in both centrilobular and portal veins, increased ballooned hepatocytes, mild steatosis, dilated sinusoids, peliosis, and both confluent and fragmentary coagulative necrosis (Figure 6a–a3, b3, c3, d3). The group receiving 2000 mg/kg exhibited perivenular and parenchymal liver inflammation, significant hemorrhage, hydropic degeneration, hepatocellular ballooning, and predominantly liquefactive necrosis, both confluent and fragmentary. At this dosage, severe fibrosis, characterized by type I collagen deposition, was identified by Masson’s trichrome staining (Figure 6a–a4, b4, c4, d4).

**FIGURE 6:**
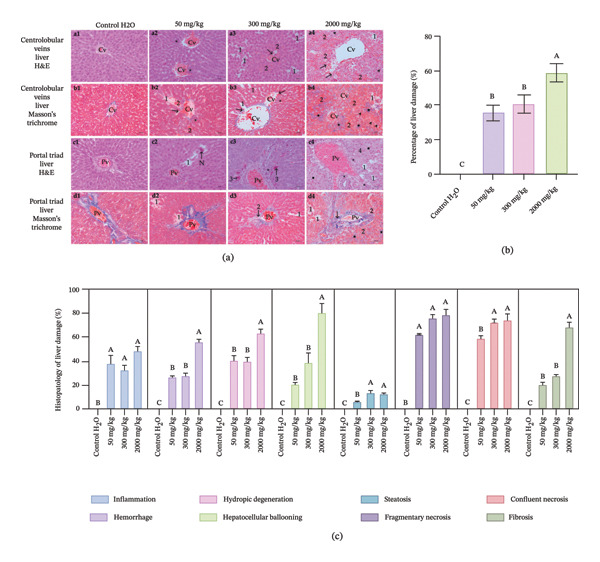
Histopathological analysis of liver tissue after oral administration of *Q. laurina* bark extract. (a) Microphotographs of liver tissue. Centrolobular vein (Cv), inflammatory cell infiltration (LPN) (asterisks), sinusoidal dilatation (1), hepatic peliosis (2), rupture of vascular endothelium (arrows), perisinusoidal fibrosis (stars), ballooning hepatocyte (arrowhead), portal vein (Pv), necrotic hepatocyte (N), acidophilic body (3), zonal necrosis (4), hydropic degeneration (point). H&E staining and Masson’s trichrome staining for evidence of fibrosis (magnification, x20). (b) Overall histopathological scoring of liver parenchyma. (c) Histopathological scoring by parameters of QLBE‐induced liver injury. The results show means ± SEM. All statistical analyses were performed using one‐way ANOVA and the nonparametric Kruskal–Wallis test, with Dunn’s post hoc test for multiple comparisons. Different letters indicate statistical significance (*p* < 0.05).

The liver damage assessment indicated a dose‐dependent detrimental effect of the QLBE on the liver Figure 6b. Analysis of histopathological scores Figure 6c revealed a statistically significant increase in hemorrhage, hydropic degeneration, hepatocellular ballooning, and fibrosis at the highest dose. Both fibrosis and hepatocellular ballooning showed a trend toward increasing severity with higher doses. These hepatic alterations underscore the severity of liver injury induced by oral QLBE administration. These changes are apparent even at the lowest 50 mg/kg dose, with significant inflammation and fragmented necrosis.

#### 3.3.2. Renal Pathology Score

Figure 7 illustrates histopathological renal damage observed after acute exposure to QLBE. The control group exhibited standard renal histological architecture (Figure 7a–e1, f1, g1, and h1). The glomerular architecture, along with the proximal and distal tubules and blood vessels, remained unchanged. At doses of 50 mg/kg QLBE, cellular adaptive changes were observed at the glomerular level, including mesangial proliferation and podocyte hypertrophy, resulting in a reduction in Bowman’s space (Figure 7a–e2, f2, g2, and h2). Some glomeruli showed atrophy of the capillary tuft. In the tubular region, copious hemorrhage and focal necrosis were observed. Treatment with 300 mg/kg QLBE (Figure 7a–e3, f3, g3, and h3) induced glomerular damage characterized by glomeruli with capillary tuft congestion, segmental sclerosis, sparse atrophy, and empty spaces without capillary tufts.

**FIGURE 7:**
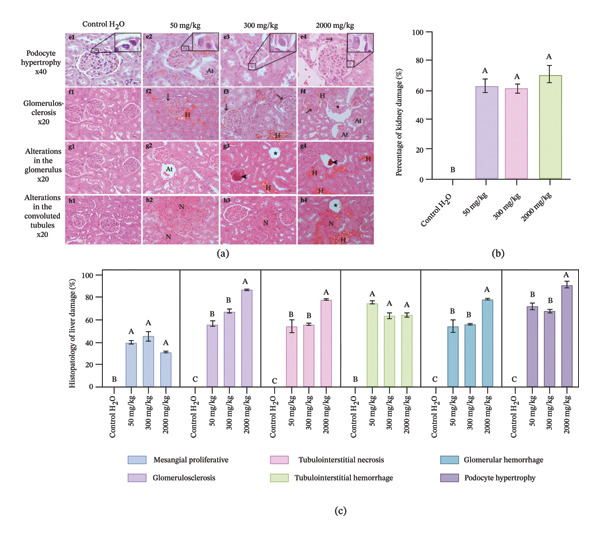
Histopathological analysis of kidney tissue after oral administration of *Q. laurina* bark extract. (a) Microphotographs of kidney tissue. Podocyte hypertrophy (inset), glomerulosclerosis (arrows), glomerular hemorrhage (arrowhead), glomerular atrophy (At), glomerulus without a capillary tuft (asterisks), tubulointerstitial hemorrhage (H), and tubulointerstitial necrosis (star). H&E staining (magnification, × 40 [a. e1, e2, e3, and e4]) and (magnification, × 20 [a. f, g, h]). (b) General histopathological scoring of the renal parenchyma. (c) Histopathological scoring by parameters of QLBE‐induced renal injury. Results are mean ± SEM. All statistical analyses were performed with one‐way ANOVA. Nonparametric Kruskal–Wallis test with Dunn’s multiple comparison post hoc test. Different letters indicate statistical significance (*p* < 0.05).

Acute oral administration of QLBE at 2000 mg/kg markedly increased kidney damage (Figure 7a–e4, f4, g4, and h4). Glomeruli showed podocyte hypertrophy, segmental sclerosis with collapse of capillary loops, and increased mesangial matrix, resulting in adhesion to the capsule. The contoured and distal tubules showed abundant necrosis and hemorrhage.

Although no significant differences were observed between doses in the overall renal histopathological scoring system, there was a trend toward increased damage at 2000 mg/kg (Figure 7b), particularly in podocyte hypertrophy, glomerulosclerosis, glomerular hemorrhage, and tubulointerstitial necrosis (Figure 7c). The occurrence of significant tubulointerstitial hemorrhage at 50 mg/kg is notable, underscoring the extent of renal injury at the lowest administered dose. These findings confirm QLBE‐induced severe nephrotoxicity after oral administration.

#### 3.3.3. Histopathology of the Brain, Heart, and Lungs

Figure 8 shows the histopathology of brain, heart, and lung tissues from organs affected by QLBE oral administration. The control group exhibited normal brain morphology (Figure 8i1). However, at 50, 300, and 2000 mg/kg, QLBE induced pia mater detachment, cerebral cortex vacuolization (also known as spongiform degeneration), necrosis of the neuropil, blood vessels with wide perivascular spaces, and mild hemorrhage (Figure 8i2, i3, i4).

**FIGURE 8:**
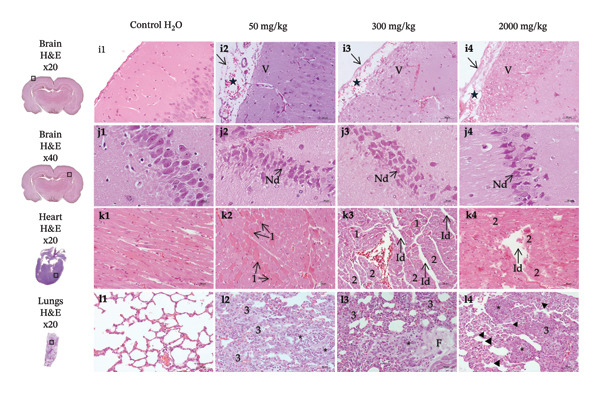
*Q. laurina* bark extract induces histopathologic alterations of brain, heart, and lung tissues after oral administration. Spongiform degeneration of cerebral cortical neuropil (V), detachment of the pia mater (arrow), hemorrhage (★), coagulative necrosis (1), liquefactive necrosis (2), interstitial edema (Id), thickening of alveolar interstitial tissue (3), inflammatory infiltrate of polymorphonuclear leukocytes (^∗^), fibrosis (F), and foamy macrophages (▲). H&E staining (magnification × 20 and × 40).

In the CA2 region of the hippocampus, the control group (Figure 8j1) exhibited pyramidal neurons with typical histological morphology. Treatment with QLBE at all administered doses induced degeneration and necrosis of hippocampal pyramidal neurons (Figure 8j2, j3, j4). These alterations were characterized by anucleated basophilic cytoplasm, perineuronal edema, narrow perivascular spaces around blood vessels, hemorrhage in the neuropil, and vacuolization. Damage was exacerbated at 2000 mg/kg.

##### 3.3.3.1. Heart

No changes in striated myocardial tissue were detected in the control group (Figure 8k1). Conversely, QLBE administration produced dose‐dependent adverse effects on the heart. Rats receiving 50 mg/kg QLBE exhibited cells with mild coagulative necrosis, characterized by pyknotic nuclei and eosinophilic cytoplasm (Figure 8k2). Additionally, areas of necrosis devoid of nuclei were identified. In the group administered 300 mg/kg, there was clear evidence of extravasated erythrocytes and interstitial edema. Myocardial cells showed a ground‐glass appearance with dense, eosinophilic, and refractile cytoplasm, indicating a more severe degree of coagulative necrosis than at the 50 mg/kg dosage. Additionally, moderate liquefactive necrosis was observed at this dose level (Figure 8k3). Animals treated with 2000 mg/kg QLBE exhibited mild hemorrhage, interstitial edema among myocardial cells, karyolysis linked to significant liquefactive necrosis, and sparse coagulative necrosis, as well as a loss of myocardiocytes (Figure 8k4).

##### 3.3.3.2. Lungs

Histopathological analysis of the lungs showed that the control group exhibited typical alveolar morphology (Figure 8l1). In contrast, oral administration of QLBE at 50, 300, and 2000 mg/kg induced significant histopathological alterations at the alveolar level, characterized by extensive inflammation, predominantly involving PMN and macrophages (Figure 8l2, l3, l4). At 300 mg/kg, collapse of the alveoli and thickening of the interalveolar septa, accompanied by fibrotic changes in the alveolar parenchyma, were observed (Figure 8l3). Hyper‐reactive macrophages within the collapsed alveoli were detected at 2000 mg/kg.

### 3.4. Effect of *Q. laurina* Bark Extract on Mitochondrial Bioenergetics in Primary Hepatocytes

Since the liver was the primary target organ affected by QLBE, the effect of the QLBE extract on mitochondrial bioenergetics and the glycolytic capacity of primary hepatocytes was evaluated to determine whether the extract alters cellular energy metabolism. A mitochondrial study was conducted on a stress test on primary hepatocytes exposed to escalating concentrations of the extract for 2 h (acute treatment, I) and 24 h (chronic treatment, II). During the acute treatment, high concentrations (1000 and 1500 μg/mL) significantly reduced basal, ATP‐linked, and maximal respiration, as well as spare respiratory capacity (Figure 9Aa, b). Additionally, basal glycolysis and glycolytic capacity were diminished (Figure 9A‐c, d). During chronic treatment, all concentrations reduced basal, ATP‐linked, maximal, and nonmitochondrial respiration and increased proton leak (Figure 9B‐a, b). Furthermore, all concentrations significantly lowered basal glycolysis, glycolytic reserve, glycolytic capacity, and nonglycolytic acidification (Figure 9B‐c, d).

**FIGURE 9:**
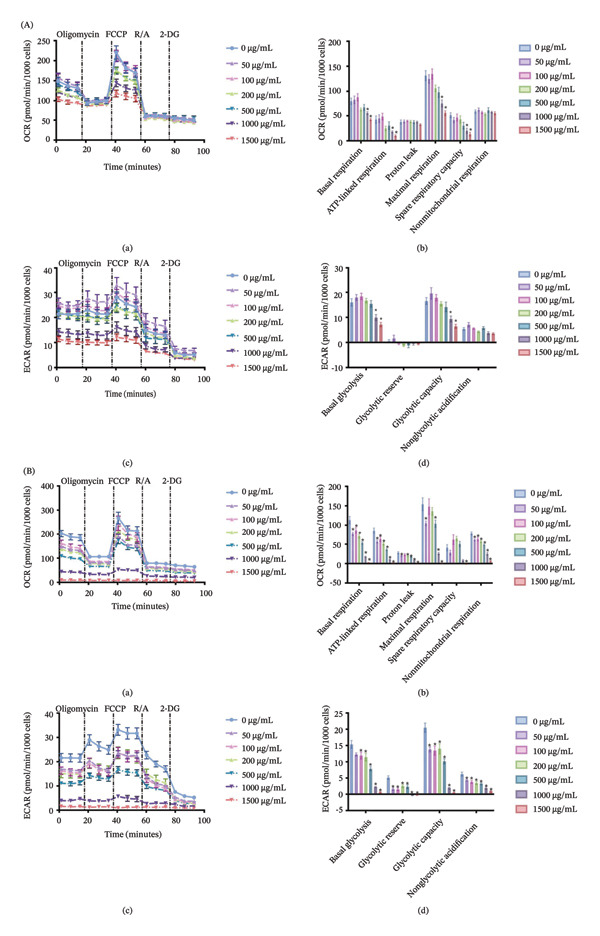
Effects of QLBE on the mitochondrial bioenergetic and glycolytic capacity of primary hepatocytes. (A) (2h of exposure), (B) (24h of exposure). (a) Oxygen consumption rate (OCR), (b) mitochondrial function parameters, (c) extracellular acidification rate (ECAR), and (d) glycolytic parameters of primary hepatocytes. Data are expressed as mean ± SEM. Data were analyzed by one‐way ANOVA followed by Dunnett’s post hoc test, with comparisons to vehicle‐treated cells. All results were considered statistically significant (*p* < 0.05)^∗^.

## 4. Discussion

Nearly 50 species of *Quercus* are used to treat dental issues, gastritis, diarrhea, cough, anxiety, muscle pain, and diabetes [39]. Oak bark is one of the most widely used parts, with significant medicinal value [40], due to its antioxidant, anti‐inflammatory, antibacterial, and cytotoxic properties [41].

Leaves, acorns, and bark of oak trees are rich in phenolic compounds [41–47]. In particular, *Quercus* bark has been considered a source of phenolic acids and hydrolyzable tannins [41], including gallic and ellagic acids, which are prominent phenolic acids in oak bark from various geographical regions. Ellagic acid, gallic acid, 3,4‐dihydroxybenzoic acid, and ferulic acid, identified by HPLC‐DAD analysis, have been previously reported in methanolic extracts of *Quercus robur*, *Quercus macrocarpa*, and *Quercus acutissima* bark [46]. Similarly, high concentrations of gallic acid have been reported in the acetone‐aqueous extract (70%) of *Q. sideroxyla* bark [48] and in the root bark of *Quercus aegilops* [49].

The elemental composition of *Q. laurina* extracts remains unknown despite its significance in traditional Mexican medicine. The carbon‐to‐hydrogen ratio suggests that the plant is rich in carbohydrates, recognized as essential constituents of cell walls. This result is consistent with GC–MS analysis, which shows that the most abundant compounds were sugars and cyclitols.

QLBE has high carbon (50.33%), oxygen (44.69%), and hydrogen (4.91%) contents and a low nitrogen content (0.07%). A higher percentage of carbon (63.90%), hydrogen (7.41%), and nitrogen (0.40%) and a lower percentage of oxygen (28.21%) were previously reported for *Quercus variabilis* cork [50]. Moreover, *Q. suber* cork also has higher carbon (61.0%) and hydrogen (8.7%) contents [51] than those found in *Q. laurina* extract, which could be related to variations in environmental factors among these species, including geographic origin, agronomic conditions, abiotic stress, stage of development, and harvest season [52].

This research, for the first time, describes the volatile and semivolatile compounds present in QLBE. To improve the analysis, QLBE was derivatized before GC–MS analysis. Derivatization modifies analyte properties to enhance separation and chromatographic selectivity, resulting in sharper peaks [53]. A total of 21 compounds were identified, most of which were classified as sugars (43%) and cyclitols (19%). A high carbohydrate content has been reported in *Quercus* acorns [54]. Species in the *Quercus* genus, such as *Q. pyrenaica, Q. alba, Q. macrocarpa, Q. robur, Q. suber, Q. ilex*, and *Q. cerris*, exhibit a distinctive cyclitol profile, which is considered a chemical marker of this genus [55].

Myo‐inositol was the primary compound detected in *Q. laurina* extract. This molecule has been previously identified in other *Quercus* species, including *Q. ilex* bark [56], *Q. pyrenaica* wood [55], and *Q. variabilis* acorns [54]. Myo‐inositol is recognized as an insulin‐sensitizing compound and is commonly used to treat polycystic ovary syndrome [57]. Although it has many pharmacological applications, there is a dearth of comprehensive data on its safety at high doses. Quininic acid and scyllo‐inositol are two other substances identified in QLBE. Extracts from *Q. pyrenaica* [55], *Q. alba, Q. petraea, and Q. robur* [58] have been shown to contain these two molecules.

The acute toxicity of QLBE administered orally in Wistar rats was evaluated to establish doses for chronic administration in subsequent studies, suggesting the use of doses below 50 mg/kg bw. Oral administration of QLBE induced lethargy, exophthalmia, and death in 50% of animals at 2000 mg/kg bw. The Globally Harmonized System of Classification and Labeling of Chemicals (GHS) classifies substances into five hazard categories based on oral LD_50_ values in animals. The criteria consist of hazard levels assigned to the five categories: I: 0–5, II: 5–50, III: 50–300, IV: 300–2000, and V: 2000–5000 mg/kg bw [59]. Consequently, QLBE was classified in Category IV according to the GSH with an LD50 of > 300–2000 mg/kg bw, as established in Annex 2c of OECD Guideline 423 [26].

Most *Quercus* species in Europe and North America are considered toxic to cattle, goats, sheep, and horses [60]. Consumption of *Q. calliprinos* leaves and acorns by steers resulted in 83% mortality, whereas ingestion of young and immature *Q. incana* leaves caused mortality in 70% of cattle [61]. A 30% mortality rate was reported in 1‐ to 2‐year‐old cattle intoxicated by *Q. robur* leaves and acorns [62]. The tendency toward increased kidney and lung weights at the highest dose indicated potential organ damage, a finding later corroborated by histopathological analysis.

Bulls ingesting 10 kg of young *Q. pyrenaica* leaves over 3 days developed gastrointestinal ulcers and renal tubular necrosis, which the authors attributed to tannins [63]. This is consistent with findings by Doce et al. (2009), who reported that the total tannin content in young *Q. pyrenaica* leaves was 230 g of tannic acid equivalents/kg of dry matter (DM) (determined via the Folin–Ciocalteu method). Furthermore, their analysis of hydrolyzable tannins using ion‐pair high‐performance liquid chromatography revealed 11 and 19 g/kg DM as gallic and ellagic acid, respectively [64]. Although veterinary toxicology extensively documents oak toxicity, these findings cannot be directly extrapolated to humans. This limitation complicates risk assessment for *Quercus* species, despite their widespread use in traditional medicine.

Results from biochemical and hematological analyses suggested that oral administration of QLBE elicits toxicity. The significant increase in leukocyte count at 2000 mg/kg may reflect tissue inflammation [65]. Leukocytosis has been reported in cattle after ingestion of *Q. pyrenaica* leaves [63] and *Quercus* spp. acorns [66]. The trend toward higher platelet levels at 2000 mg/kg (*p* = 0.08) was associated with acute inflammation [67]. Through interactions with the vascular endothelium, platelets contribute to inflammation. Platelets produce platelet‐derived growth factor and vascular endothelial growth factor after tissue damage, which are crucial instigators of inflammation [68]. Furthermore, elevated platelet counts have been associated with cardiovascular damage [69] and acute kidney injury [70].

The trend toward decline in ALT and AST levels after QLBE administration at 2000 mg/kg could suggest liver and kidney damage. In fact, a study of patients with chronic kidney disease found a direct correlation between reduced glomerular filtration rate (GFR) and lower levels of aminotransferases ALT and AST, even in patients taking potentially hepatotoxic drugs such as statins [71]. A decrease in serum ALT activity may indicate a loss of viable hepatocytes caused by necrosis in chronic liver disease or severe toxicity. This is because, after significant acute hepatocellular necrosis, serum ALT levels typically increase sharply within 24–48 h, reaching 100 times or more above normal, and peak within the first 5 days after injury [72]. However, because the half‐life of circulating ALT is about 47 h [73], serum levels decline rapidly once cell damage ceases [74]. As the extract was given as a single dose and serum ALT was measured up to Day 14, it is thought that the decrease is linked to hepatocyte necrosis and parenchyma loss caused by QLBE. This reduced the ability of hepatocytes to produce and release this enzyme. These results are consistent with histopathological observations of the liver and kidneys. In most cases, AST activity parallels changes in ALT activity. AST is not only found in the liver but also in higher concentrations in the kidneys, heart, and skeletal muscles and in measurable amounts in the brain, small intestine, and spleen. In the liver, 80% of AST activity is found in the mitochondria and the remaining 20% in the cytoplasm [75, 76].

Because urea is produced by hepatic metabolism of amino acids, lower urea levels suggest reduced protein breakdown associated with liver impairment [77]. The tendency toward decreasing creatinine levels at 2000 mg/kg was also associated with liver injury, as diminished liver function reduces creatinine production. Additionally, protein‐calorie malnutrition and muscle atrophy, frequently observed in hepatic diseases, can contribute to low creatinine production [78]. Furthermore, alterations in alkaline phosphatase, lactate dehydrogenase, and bilirubin, mainly observed at 2000 mg/kg, also suggested hepatic injury.

As the primary site of xenobiotic metabolism, the liver is particularly vulnerable to damage [79]. The QLBE, mainly at the highest dose, induced severe liver damage, characterized by hemorrhage, hydropic degeneration, hepatocyte swelling, and fibrosis. Similarly, a dose‐dependent pattern of hepatic injury has been previously reported in Wistar rats following the oral administration of *Q. brantii* hydroalcoholic extract (500 and 1000 mg/kg), which manifested as vein dilation, lymphocyte infiltration, inflammation, and necrosis [11].

Toxic substances can accumulate in the renal tubules because the kidneys filter blood continuously and receive about 25% of the cardiac output [11]. QLBE administration at 2000 mg/kg induced podocyte hypertrophy, glomerulosclerosis, glomerular hemorrhage, and tubulointerstitial necrosis. These findings align with earlier research reporting kidney injury from oral intake of oak extracts. Administration of *Q. brantii* extract induced bleeding and glomerular debris at 500 mg/kg, whereas mild‐to‐moderate tubular cell degeneration with chronic inflammatory cell infiltration was reported at 1000 mg/kg [11]. Repeated oral intake of *Q. crassifolia* bark extract at 33 and 100 mg/kg caused renal damage characterized by endothelial hyperplasia of the glomerulus in the cortex [20]. Intake of 150–200 g of oak biomass from *Q. infectoria* resulted in acute tubular necrosis and significant multifocal hemorrhages in the renal cortex of sheep [80]. Renal damage, characterized by moderate acute tubular necrosis, degenerative changes in epithelial cells, and inflammation, was previously reported in cattle fed *Q. pyrenaica* leaves [63].

QLBE‐induced brain injury suggests that some of its primary metabolites cross the blood–brain barrier (BBB), thereby causing deleterious effects. Previous studies have shown that molecules identified by GC–MS and HPLC analysis, such as stearic acid, palmitic acid [81], myo‐inositol [82], xylitol [83], t‐ferulic acid [84], and gallic acid [84], can cross the BBB. However, no reports indicate that these isolated compounds cause brain injury. Nevertheless, additional research is necessary to determine whether their combined effects might contribute to brain toxicity.

The deleterious effects of *Quercus* extracts on the brain have been poorly described. Oral administration of a purified extract of *Q. crassifolia* bark at 300 mg/kg caused brain damage in Wistar rats [85]. Consistent with histopathological findings associated with oral QLBE administration, a captive female beluga whale that consumed 20 pounds of white oak *Q. macrocarpa* developed necrotizing vasculitis of the meninges near the cortex and a hematoma in the left parietal lobe of the brain [86].

The toxicological effects of QLBE in cardiac and pulmonary tissues were like those observed with *Q. brantii* extract, which induced myocardial cell atrophy and nuclear degeneration at 50 mg/kg. In contrast, hemorrhage and necrosis were observed at 500 and 1000 mg/kg [11]. *Q. brantii* extract provoked eosinophil and inflammatory cell infiltration in the lungs at 500 and 1000 mg/kg, which aligns with the histological alterations induced by QLBE at 300 and 2000 mg/kg [11].

Hydrolyzable tannins, particularly gallotannins and their derivatives, commonly found in *Quercus* extracts, may contribute to toxicity [87]. Bacterial breakdown of these tannins produces digallic acid, which is then converted to gallic acid and pyrogallol [88]. Both pyrogallol and gallic acid induce severe renal damage, including acute tubular necrosis, hyperplasia, and uremia [89]. In addition, pyrogallol induces hepatotoxicity via oxidative stress [90], while subchronic dietary exposure to gallic acid (≥ 0.6%–5% in the diet) causes hemolytic anemia, centrilobular hepatocyte hypertrophy, and proximal tubular pigment accumulation [91]. Furthermore, acute intraperitoneal administration of gallic acid triggers severe diffuse neuronal degeneration and neuropil loss in rat brains [92]. Whether taken separately or in combination, gallic and ellagic acids have been shown to cause renal damage, including hyperplasia, juxtaglomerular apparatus necrosis, and acute tubular necrosis [22]. Consequently, the gallic and ellagic acids identified in QLBE via HPLC and GC–MS could be related to the observed hepatotoxic and nephrotoxic effects.

Given the severity of hepatic damage caused by QLBE exposure and the liver’s crucial role in the first‐pass metabolism of xenobiotics [93, 94], further investigation into the effects of QLBE on primary hepatocytes’ bioenergetics was conducted. The study specifically examined how QLBE affected mitochondrial bioenergetics and glycolytic capacity of hepatocytes across different concentrations and exposure times. The evaluation in primary hepatocytes complements the in vivo observations, since whole‐animal models do not always allow discrimination between direct cellular toxicity and secondary systemic effects.

Mitochondrial toxicants can reduce the OCR and basal respiration while altering the ECAR. OCR measures mitochondrial respiration and plays a crucial role in ATP production in aerobic organisms. In contrast, ECAR is a marker of glycolysis [95, 96]. Simultaneous measurement of OCR and ECAR using technologies such as the Seahorse extracellular flux analyzer, using specific compounds such as oligomycin, rotenone, and antimycin titrated to inhibit specifically diverse complexes of the oxidative phosphorylation pathway, allows for accurate assessment of alterations in mitochondrial function and cellular energy metabolism induced by bioactive and toxic compounds [97]. In this context, changes in OCR together with alterations in ECAR provide evidence of disrupted cellular bioenergetics induced by the QLBE extract in a dose‐ and time‐dependent manner. However, the present study does not establish whether mitochondrial dysfunction directly mediates the hepatotoxic effects observed in vivo.

After two hours of exposure, corresponding to the acute treatment phase, the highest QLBE concentrations (1000 and 1500 μg/mL) significantly reduced basal, ATP‐linked, and maximal respiratory capacities, spare respiratory capacity, basal glycolysis, and glycolytic capacity. Following 24 h of exposure, representing a chronic in vitro exposure, alterations in mitochondrial and glycolytic parameters were observed at lower concentrations, suggesting that the effects of QLBE on hepatocyte bioenergetics depend on both dose and exposure duration. Determining both acute and chronic exposure for toxicological assessment provides evidence of the early metabolic alterations preceding more pronounced cellular dysfunction during sustained exposure.

A limitation of the present study is that a dedicated viability study was not performed to determine whether the observed bioenergetic alterations at the highest concentration may partially reflect reduced cellular viability. However, nuclear counts obtained by Hoechst staining at the end of the mitochondrial stress test did not differ among treatments at either exposure time, suggesting that the observed changes in OCR and ECAR were not attributable to differences in cell number. Therefore, these results indicate that QLBE can disrupt hepatocyte energy metabolism in a manner dependent on concentration and exposure time, although additional studies will be required to determine the extent to which these alterations reflect direct mitochondrial toxicity or secondary effects associated with cytotoxicity.

It should be noted that the significant effect of QLBE at 1000 and 1500 μg/mL in reducing SRC began within two hours of administration. Among the various mitochondrial parameters, SRC stands out for its low variability, reproducibility, and high sensitivity in assessing mitochondrial adaptation [98]. This parameter is calculated by subtracting basal respiration from the peak level of mitochondrial respiration. Because mitochondrial function is highly dynamic, cells can mobilize SRC as needed. Consequently, SRC reflects mitochondria’s ability to meet increased energy demands beyond the baseline level in response to acute cellular stress, such as toxic damage, thereby averting ATP deficiency. Therefore, SRC is considered a marker of “healthful” mitochondria [98].

SRC varies with the cell type and is influenced by the activity of respiratory chain components [98]. Given that previous reports have shown that inhibiting complexes II and IV reduces SRC levels in cardiomyocytes [99, 100], it is possible to hypothesize that the effects of QLBE on SRC could be partially mediated by its impact on the respiratory chain complexes. Oral administration of gallic acid to Wistar rats has been shown to markedly decrease the activities of complexes II and IV in kidney mitochondria [22]. Therefore, its presence in the QLBE, as shown by the GC–MS analysis, could be associated with the SRC diminution in primary hepatocytes.

The evolution of liver damage is linked to alterations in mitochondrial bioenergetics [101]. The extent of liver injury determines the severity of these harmful effects, which include necrosis, fibrosis, and liver inflammation. Patients with mild to moderate liver fibrosis have shown an increase in SRC compared with healthy individuals [101]. However, SRC drops markedly in patients with severe liver fibrosis compared with those with mild fibrosis. Furthermore, compared with people with mild hepatic fibrosis, those with severe fibrosis exhibit a significant decrease in their maximal, ATP‐linked, and basal respiratory capacities [101]. QLBE at 2 h of exposure (1000 and 1500 μg/mL) induces a significant decrease in basal, ATP‐linked, and maximal respiratory capacity in hepatic mitochondria, which could correlate with the severe fibrosis observed in histopathological analysis.

The reduction in basal glycolysis and glycolytic capacity triggered by QLBE is remarkable and evident within 2 hours of administration. These effects persist over 24 h. In normal cells, oxidative phosphorylation is the primary source of ATP. However, under stress conditions, such as toxic exposure, cells shift between predominantly oxidative phosphorylation and glycolysis, depending on the microenvironmental characteristics [102]. It has been reported that during the initial stages of liver failure, mitochondrial function and ATP synthesis are preserved by increasing energy production through glycolysis as oxidative phosphorylation output declines. However, in the later stages of liver injury, mitochondrial respiration and ATP production are severely impaired, and hepatocytes′ glycolytic activity cannot meet the increased demand for ATP. In this scenario, the inability to carry out glycolysis is a hallmark of hepatocyte failure in advanced liver damage [102].

Hepatocyte injury triggers cellular swelling as part of the necrotic process, a histopathological feature that ultimately leads to cell membrane rupture. The rupture releases intracellular components, thereby triggering secondary inflammation. This leads to liver insufficiency, which impairs synthetic function, interferes with intrahepatic metabolism, and reduces the ability to remove circulating cytokines, all of which affect the adequate function of other organs such as the kidneys, lungs, and brain, leading to multisystemic involvement [93, 94].

Overall, this research demonstrates for the first time that QLBE induces significant liver, kidney, brain, heart, and lung damage at doses as low as 50 mg/kg. This dose corresponds to a human equivalent dose (HED) of 8.06 mg/kg, which translates to approximately 564 mg of extract for a 70 kg individual. Considering the extraction yield of 16.84% obtained in this study, this amount of extract is equivalent to approximately 3.35 g of dry bark. Consequently, the oral ingestion of traditional preparations delivering this raw material equivalent could pose a toxicological risk to consumers.

Unfortunately, data regarding the precise exposure levels or preparation methods of *Q. laurina* in the Mexican population remain unavailable. However, the empirical use of *Quercus* bark, as described in traditional herbal medicine monographs, suggests its consumption as an infusion (2–4 g in 150 mL of water, 2–3 times daily) and as a tincture (1:5, 40% ethanol; 2–5 mL, 2–3 times daily) [103]. Notably, these traditional infusion dosages (2–4 g) directly overlap with the 3.35 g dry bark equivalent calculated to induce acute toxicity in this investigation.

Furthermore, previous studies on other species belonging to the *Quercus* genus have reported traditional or commercial oral dosages that underscore this risk. For instance, *Q. brantii* extract has been administered at 300 mg/day for 3 months [104], *Q. robur* extract (Robuvit) is used at 300–600 mg/day [105], and *Q. salicina* has been documented at 450 mg three times daily [106]. Although the therapeutic dosages of related *Quercus* species cannot be directly extrapolated to predict the toxicity of *Q. laurina* due to inherent variations in phytochemical profiles, extraction procedures, and treatment durations, they provide a valuable benchmark for commercial exposure levels. Notably, the fact that these common commercial doses closely align with the 564 mg threshold, where toxic effects were empirically detected in the present study, raises safety concerns regarding the traditional use of *Q. laurina*.

This toxicological concern is particularly relevant considering the current commercial landscape in Mexico. Currently, various parts of oak trees are widely accessible to the public through traditional street markets and online platforms such as Amazon and “Mercado Libre.” Consequently, the informal sale of these materials could pose significant health hazards to consumers. This risk is heightened by the ease of public access and the lack of stringent regulations for herbal remedies and dietary supplements in Mexico, where such products are not required to demonstrate scientific evidence of safety or therapeutic efficacy, relying solely on traditional or popular knowledge [107]. Therefore, further repeated‐dose toxicity studies are essential to establish the no‐observed‐adverse‐effect level (NOAEL) and the lowest‐observed‐adverse‐effect level (LOAEL) for QLBE, which will allow for a precise risk characterization of its oral exposure in the Mexican population.

## 5. Conclusions

In summary, this research demonstrated that QLBE exposure is associated with severe liver injury and alterations in hepatocyte energy metabolism. The in vitro experiments in primary hepatocytes showed that QLBE impairs mitochondrial bioenergetics and glycolytic capacity in a dose‐ and time‐dependent manner, suggesting that altered cellular energy metabolism may contribute to the hepatotoxic effects observed in vivo. However, the present study does not establish a direct causal relationship between mitochondrial dysfunction and liver injury, and further studies will be required to clarify the mechanism involved.

Oral administration of QLBE produced dose‐dependent hepatic injury characterized by severe fibrosis and necrosis, consistent with advanced liver damage. In addition, acute liver failure induced by QLBE was associated with pathological alterations in other organs, including the brain, kidneys, heart, and lungs, indicating multisystemic toxicity.

Because QLBE elicits hepatic, renal, brain, pulmonary, and cardiac toxicity at doses as low as 50 mg/kg, which corresponds to a HED of 8.06 mg/kg (approximately 564 mg for a 70 kg individual), caution is warranted when consuming this extract orally in traditional medicine remedies. Further investigations are needed to identify the bioactive compounds or metabolites responsible for the toxic effects. In addition, subacute, subchronic, and chronic toxicological studies are required to characterize the safety profile of QLBE following prolonged and repeated oral exposure.

## Author Contributions

Conceptualization: Martha‐Estrella García‐Pérez and Rosa‐E. del Río; methodology: Grecia‐Elena Hurtado‐Nuñez, Sandra‐Guadalupe Sánchez‐Ceja, Lilia‐Guadalupe Noriega, Sandra Tobón‐Cornejo, José‐Juan Virgen‐Ortíz, Yoshio‐de‐Jesús Mena‐Gavia, Héctor‐Eduardo Martínez‐Flores, Rosa‐E. del Río, and Martha‐Estrella García‐Pérez; software, Grecia‐Elena Hurtado‐Nuñez, Lilia‐Guadalupe Noriega, Sandra Tobón‐Cornejo, José‐Juan Virgen‐Ortíz, Yoshio‐de‐Jesús Mena‐Gavia, and Jorge‐Armando Tinoco‐Michel; validation, Lilia‐Guadalupe Noriega, Sandra Tobón‐Cornejo, José‐Juan Virgen‐Ortíz, María‐Carmen Bartolomé‐Camacho, and Rosa‐E. del Río; formal analysis, Grecia‐Elena Hurtado‐Nuñez, Sandra‐Guadalupe Sánchez‐Ceja, Lilia‐Guadalupe Noriega, Sandra Tobón‐Cornejo, Isis‐Beatriz Bermúdez‐Camps, María‐Carmen Bartolomé‐Camacho, Héctor‐Eduardo Martínez‐Flores, and Martha‐Estrella García‐Pérez; investigation, Grecia‐Elena Hurtado‐Nuñez, Sandra‐Guadalupe Sánchez‐Ceja, Lilia‐Guadalupe Noriega, Sandra Tobón‐Cornejo, and Rosa‐E. del Río; resources, Sandra‐Guadalupe Sánchez‐Ceja, Lilia‐Guadalupe Noriega, José‐Juan Virgen‐Ortíz, Héctor‐Eduardo Martínez‐Flores, and Martha‐Estrella García‐Pérez; data curation, Grecia‐Elena Hurtado‐Nuñez, Sandra‐Guadalupe Sánchez‐Ceja, Lilia‐Guadalupe Noriega, Sandra Tobón‐Cornejo, José‐Juan Virgen‐Ortíz, Yoshio‐de‐Jesús Mena‐Gavia, Isis‐Beatriz Bermúdez‐Camps, María‐Carmen Bartolomé‐Camacho, Héctor‐Eduardo Martínez‐Flores, Rosa‐E. del Río, and Martha‐Estrella García‐Pérez; writing–original draft preparation, Grecia‐Elena Hurtado‐Nuñez; writing–review and editing, Sandra‐Guadalupe Sánchez‐Ceja, Lilia‐Guadalupe Noriega, Sandra Tobón‐Cornejo, José‐Juan Virgen‐Ortíz, Yoshio‐de‐Jesús Mena‐Gavia, Jorge‐Armando Tinoco‐Michel, Isis‐Beatriz Bermúdez‐Camps, María‐Carmen Bartolomé‐Camacho, Héctor‐Eduardo Martínez‐Flores, Rosa‐E. del Río, and Martha‐Estrella García‐Pérez; visualization, Grecia‐Elena Hurtado‐Nuñez, Sandra‐Guadalupe Sánchez‐Ceja, Lilia‐Guadalupe Noriega, and Martha‐Estrella García‐Pérez; supervision, Sandra‐Guadalupe Sánchez‐Ceja, Lilia‐Guadalupe Noriega, José‐Juan Virgen‐Ortíz, Isis‐Beatriz Bermúdez‐Camps, María‐Carmen Bartolomé‐Camacho, Héctor‐Eduardo Martínez‐Flores, Rosa‐E. del Río, and Martha‐Estrella García‐Pérez; project administration, Martha‐Estrella García‐Pérez, and Rosa‐E. del Río; funding acquisition, Martha‐Estrella García‐Pérez.

## Funding

The authors would like to thank Mexico’s Secretariat of Science, Humanities, Technology, and Innovation (SECIHTI) for the doctoral fellowship awarded to Grecia‐Elena Hurtado‐Nuñez (No. 735795), which provided financial support for this research. The work also benefited from institutional resources and collaboration from the Universidad Michoacana de San Nicolás de Hidalgo, the Michoacán Center for Agri‐Food Innovation and Development (CIDAM, AC), and the Salvador Zubirán National Institute of Medical Sciences and Nutrition.

## Disclosure

All authors have read and agreed to the published version of the manuscript.

## Conflicts of Interest

The authors declare no conflicts of interest.

## Supporting Information

Additional supporting information can be found online in the Supporting Information section.

## Supporting information


**Supporting Information** Table 1S: Histopathology score of hepatic lesions; Table 2S: Renal pathology score.

## Data Availability

Data are available on request from the authors.
