# Supplementary material for: Toxicological Profile of a Quercus laurina Bark Extract Through Oral Administration: Impact on Hepatocyte Mitochondrial Bioenergetics
Source: J Toxicol. 2026 Aug 17;2026:8100007. doi: 10.1155/jt/8100007 (PMC13481712; doi:10.1155/jt/8100007)
Supplement: Supplementary file 1 [file JT-2026-8100007-s001.docx]

*Supplementary material*

Toxicological profile of a *Quercus laurina* bark extract through oral administration: impact on hepatocyte mitochondrial bioenergetics

**Table 1S.** Histopathology score of hepatic lesions

| Evaluation index | Degree | Points | Reference |
| --- | --- | --- | --- |
| a) Portal inflammation | Absent | 0 | [1] |
|  | Mild | 1 |  |
|  | Moderate | 3 |  |
|  | Marked | 4 |  |
| b) Hemorrhage | No hemorrhage | 0 | - |
|  | Mild | 1 |  |
|  | Moderate | 2 |  |
|  | Marked | 3 |  |
| c) Hydropic degeneration | Absent | 0 | [2] |
|  | Mild | 1 |  |
|  | Moderate | 2 |  |
|  | Marked | 3 |  |
| d) Steatosis | No steatosis | 0 | [2] |
|  | Mild | 1 |  |
|  | Moderate | 2 |  |
|  | Marked | 3 |  |
| e) Ballooning | Absent | 0 | [2] |
|  | Mild | 1 |  |
|  | Moderate | 2 |  |
|  | Marked | 3 |  |
|  | No |  |  |
| f) Fragmentary necrosis | Absent | 0 | [1] |
|  | Mild (focal, few portal  areas) | 1 |  |
|  | Mild/moderate (focal,  most portal areas) | 2 |  |
|  | Moderate (continuous around <50% of tracts or septa) | 3 |  |
|  | Severe (continuous around ＞50% of tracts or septa) | 4 |  |
| g) Confluent necrosis | Absent | 0 | [1] |
|  | Focal confluent necrosis | 1 |  |
|  | Zone 3 necrosis in some  areas | 2 |  |
|  | Zone 3 necrosis in most  areas | 3 |  |
|  | Zone 3 necrosis + occasional portal-central (P-C) bridging | 4 |  |
|  | Zone 3 necrosis +  multiple P-C bridging | 5 |  |
|  | Panacinar or multiacinar necrosis | 6 |  |
| h) Fibrosis | No fibrosis | 0 | [1] |
|  | Fibrous expansion of some portal areas, with or without short fibrous septa | 1 |  |
|  | Fibrous expansion of most portal areas, with or without short fibrous septa | 2 |  |
|  | Fibrous expansion of most portal areas with occasional portal to portal (P-P) bridging | 3 |  |
|  | Fibrous expansion of portal areas with marked bridging [portal to portal (P-P) as well as portal to central (P-C)] | 4 |  |
|  | Marked bridging (P-P and/or P-C) with occasional nodules (incomplete cirrhosis) | 5 |  |
|  | Cirrhosis, probable or definite | 6 |  |

With modifications: include inflammation by polymorphonuclear leukocytes, hemorrhage and hepatocellular ballooning.

The scoring criteria for renal lesions were established as described by Liu *et al.,* 2020 [3], with modifications (**Table 2S**).

**Table 2S.** Renal pathology score criteria.

| Evaluation index | Degree | Points |
| --- | --- | --- |
| a) Glomerular mesangial proliferation | No mesangial proliferation | 0 |
|  | Mild mesangial proliferation | 1 |
|  | Moderate mesangial proliferation | 2 |
|  | Severe mesangial proliferation | 3 |
| b) Degree of glomerular sclerosis | No glomerular sclerosis | 0 |
|  | Glomerular sclerosis rate <25% | 1 |
|  | Glomerular sclerosis rate 25–50% | 2 |
|  | Glomerular sclerosis rate >50% | 3 |
| c) Podocyte hypertrophy | No podocyte hypertrophy | 0 |
|  | Podocyte hypertrophy rate <25% | 1 |
|  | Podocyte hypertrophy rate 25–50% | 2 |
|  | Podocyte hypertrophy rate >50% | 3 |
| d) Glomerular capillary congestion (hemorrhage) | No glomerular hemorrhage | 0 |
|  | Glomerular hemorrhage rate <25% | 1 |
|  | Glomerular hemorrhage rate 25–50% | 2 |
|  | Glomerular hemorrhage rate >50% | 3 |
| e) Renal tubulointerstitial score (RTIS) was determined according to four indicators: (1) degeneration and necrosis of renal tubule; (2) tubular atrophy; (3) infiltration of interstitial inflammatory cells; (4) hemorrhage tubulointerstitial (5) interstitial fibrosis. | No severity of lesions | 0 |
|  | Severity of lesions <25% | 1 |
|  | Severity of lesions 25–50% | 2 |
|  | Severity of lesions >50% | 3 |

With modifications: include podocyte hypertrophy, glomerular capillary congestion, and hemorrhage tubulointerstitial.

References

1.	Son MS, Yoo JH, Kwon CI, Ko KH, Hong SP, Hwang SG, et al. Associations of expressions of HBcAg and HBsAg with the histologic activity of liver disease and viral replication. Gut Liver. 2008 Dec;2(3):166–73. doi:10.5009/gnl.2008.2.3.166 PubMed PMID: 20485642; PubMed Central PMCID: PMC2871644.

2.	Caballería Rovira L, Majeed I, Martínez Escudé A, Arteaga Pillasagua I, Torán Monserrat P. Esteatosis hepática: diagnóstico y seguimiento. FMC - Formación Médica Continuada en Atención Primaria. 2017 Aug;24(7):378–89. doi:10.1016/j.fmc.2017.03.001

3.	Liu W, Li Y, Xiong X, Chen Y, Qiao L, Wang J, et al. Traditional Chinese medicine protects against hypertensive kidney injury in Dahl salt-sensitive rats by targeting transforming growth factor-β signaling pathway. Biomedicine & Pharmacotherapy. 2020 Nov 1;131:110746. doi:10.1016/j.biopha.2020.110746
